# Life on the edge: behavioural and physiological responses of Verreaux's sifakas (*Propithecus verreauxi*) to forest edges

**DOI:** 10.5194/pb-8-1-2021

**Published:** 2021-02-09

**Authors:** Klara Dinter, Michael Heistermann, Peter M. Kappeler, Claudia Fichtel

**Affiliations:** 1 Behavioral Ecology and Sociobiology Unit, German Primate Center, Kellnerweg 4, 37077 Göttingen, Germany; 2 Endocrinology Laboratory, German Primate Center, Kellnerweg 4, 37077 Göttingen, Germany; 3 Department of Sociobiology and Anthropology, Johann-Friedrich-Blumenbach Institute for Zoology and Anthropology, Georg-August University, Kellnerweg 6, 37077 Göttingen, Germany

## Abstract

Forest edges change micro-environmental conditions, thereby affecting the ecology of many forest-dwelling species. Understanding such edge effects is particularly important for Malagasy primates because many of them live in highly fragmented forests today. The aim of our study was to assess the influence of forest edge effects on activity budgets, feeding ecology, and stress hormone output (measured as faecal glucocorticoid metabolite – fGCM – levels) in wild Verreaux's sifakas (*Propithecus verreauxi*), a group living, arboreal lemur. We observed five habituated groups: three living in the forest interior and two at an established forest edge. There was no difference in average daily temperatures between edge and interior habitats; however, within the edge site, the average daily temperature incrementally increased over 450 m from the forest edge towards the interior forest of the edge habitat, and the population density was lower at the edge site. Activity budgets differed between groups living in the two microhabitats, with individuals living near the edge spending more time travelling and less time feeding. Groups living near the edge also tended to have smaller home ranges and core areas than groups in the forest interior. In addition, edge groups had elevated average fGCM concentrations, and birth rates were lower for females living in the edge habitat. Combined with lower levels of fruit consumption at the edge, these results suggest that nutritional stress might be a limiting factor for Verreaux's sifakas when living near a forest edge. Hence, Verreaux's sifakas appear to be sensitive to microhabitat characteristics linked to forest edges; a result with implications for the conservation of this critically endangered lemurid species.

9 February 2021

## 1 Introduction

Tropical forest habitats continue to become increasingly fragmented, thereby disintegrating populations and reducing suitable habitats for many species (Laurance, 1999; Watson et al., 2004). Fragmentation of habitats results in additional abrupt boundaries, creating so-called edge effects. Edge effects refer to how abiotic and biotic conditions interact between two adjacent habitats separated by an abrupt boundary, altering the distribution, abundance, and behaviour of organisms (Murcia, 1995). As environmental conditions, such as temperature, light intensity, plant growth, and level of disturbance, change around edges, they can be considered as distinct ecosystems in comparison to undisturbed forest interiors (Laurance et al., 2000, 2011). Species responses towards edges are diverse (Lidicker, 1999): species that exhibit their highest densities at the edge are considered to show an edge-positive response, whereas species that avoid forest edges are considered to exhibit an edge-negative response, and species that show little or no response towards the edge are considered to be edge-neutral. Because species respond differently to living in edge habitats (Lehtinen et al., 2003; Lehman et al., 2006a, b; Delattre et al., 2009), studying edge effects is of key importance for species-specific conservation assessment.

The effects of anthropogenic habitat changes are manyfold. First, numerous studies have demonstrated that disturbed environments are associated with elevated stress hormone, i.e. glucocorticoid (GC), levels in many vertebrate taxa, including primates (Dantzer et al., 2014). Specifically, in Yucatan spider monkeys (*Ateles geoffroyi yucatanensis*; Rangel-Negrín et al., 2009), brown spider monkeys (*Ateles hybridus*; Rimbach et al., 2013a), black howler monkeys (*Alouatta pigra*; Martínez-Mota et al., 2007; Rangel-Negrín et al., 2014), and grey-cheeked mangabeys (*Lophocebus albigena*; Jaimez et al., 2012) individuals living in disturbed and fragmented forests exhibited elevated GC levels compared with those inhabiting continuous and undisturbed forest patches. Collared brown lemur (*Eulemur collaris*) also exhibited elevated GC concentrations in degraded forests, where reduced fruit availability exacerbated the ecological challenges faced by these lemurs (Balestri et al., 2014). Hence, physiological stress responses to disturbed habitats are a major focus in conservation physiology because they provide a quantitative measure of how environmental changes impact individuals (Tarlow and Blumstein, 2007; Dantzer et al., 2014). Second, habitat fragmentation had a generally negative effect on the distribution of 32 lemur species (Eppley et al., 2020). Folivorous–frugivorous species, however, showed greater flexibility and variability in terms of habitat area and landscape complexity compared with nearly exclusively folivorous or frugivorous species, which appear to exhibit some resilience to forest fragmentation (Eppley et al., 2020). Third, population density and juvenile recruitment can depend on fragment size and the availability of key feeding trees, as shown for ring-tailed lemurs (*Lemur catta*; Gould and Cowen, 2020). Finally, habitat fragmentation may eventually also reduce genetic diversity, as shown in species such as the black-and-white ruffed lemurs (*Varecia variegata*: Baden et al., 2019) and golden-brown mouse lemurs (*Microcebus ravelobensis*; Radespiel et al., 2019), leading to increased vulnerability due to stochastic demographic changes and potential bottlenecks.

As a biodiversity hotspot, Madagascar suffers from an alarming rate of habitat conversion and fragmentation (Harper et al., 2007; Schwitzer et al., 2014; Vieilledent et al., 2018). Madagascar has lost 44 % of its natural forest cover over the 1953–2014 period (including 37 % over the 1973–2014 period; Vieilledent et al., 2018). Anthropogenic influences and habitat disturbance also have vital consequences for the endemic primates of Madagascar (Lemuriformes), which are considered to be the most endangered group of mammals today (Schwitzer et al., 2014). Some lemurs exhibit a negative edge response, such as *Cheirogaleus major* (Lehman et al., 2006a–c) and *M. ravelobensis* (Andriatsitohaina et al., 2019), but others have been found to exhibit a neutral (*Eulemur rubriventer*, *Hapalemur griseus*, and *Avahi laniger*) or even positive edge response (*Microcebus rufus* and *Propithecus edwardsi*), stressing the importance of studies of individual species or populations.

Among sifakas (genus *Propithecus*: Indridae), which are folivorous and, hence, potentially less resilient to habitat fragmentation (Eppley et al., 2020), responses to edges differ between species. Coquerel's sifakas (*Propithecus coquereli*) seem to avoid edge areas and do not range right on the forest edge but within 400 m of it, resulting in larger home ranges in comparison with groups occurring in the forest interior, although food quality does not differ between habitats (McGoogan et al., 2009). Diademed sifakas (*Propithecus diadema*) that live in forest fragments have smaller home ranges and appear to avoid feeding at fragment edges in contrast to groups living in the forest interior (Irwin, 2008). Subsequent blood analyses revealed a negative edge response on the physiological health of these animals as reflected by lower values of white blood cell counts, total protein, or iron-binding capacity (Irwin et al., 2010). Moreover, morphological traits of Diademed sifakas were affected by habitat quality, with individuals living in smaller fragments with low habitat quality exhibiting “wasting effects” in adults and “stunting effects” in juveniles (Irwin et al., 2019).

Given the interspecific variation in terms of behavioural and physiological effects to living in an edge habitat, the objective of this study was to investigate how forest edge habitats influence behavioural patterns, feeding ecology, and physiological stress hormone output in Verreaux's sifakas (*Propithecus verreauxi*), which were recently classified as “Critically Endangered” (IUCN, 2020). To this end, we studied three groups of sifakas inhabiting the interior of an intact forest area and two groups living at the forest edge along a savannah (Rakotoniaina et al., 2016). To determine whether Verreaux's sifakas respond to the associated differences in habitat structure, we collected data on ambient temperature, home range size, activity budgets, diet, faecal glucocorticoid metabolite output, and reproductive rates, and we compared these variables between the two populations.

## 2 Methods

### 2.1 Study site and subjects

This study was conducted at the German Primate Center (DPZ) field station in Kirindy Forest (44${}^{\circ }$39${}^{\prime }$ E, 20${}^{\circ }$04${}^{\prime }$ S) in central western Madagascar. Data were collected from April to July 2012, which corresponds to the transition from the local wet (November–April) to dry (May–October) season. Most trees shed their leaves over the dry season, reducing food availability of the predominantly folivorous sifakas (Koch et al., 2017). The local study areas were situated about 2 km from each other: one in the forest interior and one bordering a treeless savannah that has been present for at least several decades (Rakotoniaina et al., 2016). We studied five groups with a total number of 20 individuals: three groups inhabited the forest interior and two groups lived at the forest edge close to the savannah (Table 1). Both study areas were fitted with a grid system of small foot trails (Kappeler and Fichtel, 2012).

**Table 1:** Composition of the five study groups at the two study sites (“f” denotes females, and “m” denotes males).

Site	Group	Size	Adult	Adult	Subadults	Juveniles
			females	males		
Edge	2	2	1	1	0	0
	3	6	1	3	1 (f)	1 (m)
Interior	C	4	1	2	1 (f)	0
	G	4	1	2	1 (f)	0
	L	3 (4)	1	1	1 (m)	1 (f) †

† represents that animals died or disappeared during the study.

Verreaux's sifakas are diurnal, arboreal, and live in groups of 2–12 individuals (mean ± SD: 6±2 individuals; Kappeler and Fichtel, 2012), which occupy home ranges that can vary in size due to seasonality, habitat quality, or group size (Carrai and Lunardini, 1996; Benadi et al., 2008; Rudolph et al., 2020). In the interior of Kirindy Forest, home range sizes ranged between 5 and 10 ha (Benadi et al., 2008; Koch et al., 2016), but corresponding data were not previously available for the forest edge. All study subjects were well habituated to human observers and marked individually by unique nylon collars and one radio transmitter per group (Kappeler and Fichtel, 2012).

### 2.2 Temperature measurement

Ambient temperature was measured once per hour continuously throughout the study using iButtons (1-Wire/iButton DS1921G-F5; accuracy ±1 ${}^{\circ }$C). Both study sites are equipped with a grid system with 50 m × 50 m trails in the edge habitat and 25 m × 25 m trails in the forest interior. At each site, 10 iButtons were placed at breast height on tree trunks, with a distance of 50 m between them, on a perpendicular line of the grid systems, and the forest edge (Fig. 2). In the savanna, the first iButton was placed at the border of the edge and the last iButton was placed 450 m westwards. In the forest interior, the first iButton was placed in the centre of the study area and the last iButton was placed 450 m westwards.

### 2.3 Behavioural data collection

Behaviour of sifakas was recorded using continuous focal sampling (Altmann, 1974) of 1 h duration of all adult individuals of a group, resulting in 252 h of focal animal data. Individuals were chosen as focal animals in a randomized but counterbalanced order throughout the day, and we observed all individuals equally often. Observed behavioural states were assigned to one of four categories: feeding (F), social behaviour (S), travelling (T), and resting (R). The feeding category included searching, manipulating, and ingesting food items within a food patch (i.e. tree). To characterize dietary composition, we recorded the plant parts of every species that a focal individual fed on within a feeding bout for longer than 5 min (e.g. leaves, fruits, buds, or flowers; Benadi et al., 2008; Koch et al., 2017). In addition, the GPS position of the plant was noted, the species was identified, and feeding tree characteristics such as diameter at breast height (dbh), height, and crown diameter were recorded.

The social behaviour category included all interactions between individuals, including affiliation (duration of grooming and huddling), agonism (frequency of bites and threats), and affinity (approaches within 1 m). The travelling category included all movements or changes in location of at least 5 m by jumping or climbing (Trillmich et al., 2004). Spending at least 30 s without engaging in any of the above activities (e.g. sitting, watching, autogrooming, or sleeping) was recorded as resting. To determine the home range sizes, the location of each group was additionally recorded by taking the GPS position (Garmin CS76) of the focal animal every 15 min.

### 2.4 Faecal sample collection and faecal glucocorticoid metabolite (fGCM) analysis

To measure stress hormone output via the determination of faecal glucocorticoid metabolite (fGCM) concentrations, three faecal samples per focal individual were collected between 8:00 and 10:00 UTC+3 (N=43 except seven samples that were collect between 13:00 and 14:00 UTC+3) in three different sampling sessions every 2 weeks (mean ± SD: 13±2 d) from mid-May to the end of June. Each sample consisted of exactly five faecal droplets (in total 0.5–2.2 g fresh weight) that were collected immediately after defaecation and directly transferred into labelled polypropylene tubes containing 8 mL of 80 % watery ethanol. Faeces where urine contamination was suspected were not sampled. All samples were extracted on-site on the day of sample collection, using a validated method for extracting faecal hormone metabolites under field conditions (Shutt et al., 2012).

Briefly, each sample was homogenized in its original ethanolic solvent using a metal rod, and the resulting faecal suspensions were subsequently vortexed for 2 min (Lab Dancer S40). Samples were finally centrifuged for 2 min using a manually operated centrifuge. About 1 mL of the supernatant was decanted into a labelled 2 mL polypropylene tube, sealed with PARAFILM, and stored in the dark at ambient temperature until transport to the endocrinology laboratory of the German Primate Center within 3 weeks of the end of sample collection. Thus, sample extracts were stored in the field for a maximum of 4–5 months, a period during which fGCM concentrations have been shown to remain stable (Shutt et al., 2012; Rimbach et al., 2013b; Kalbitzer and Heistermann, 2013).

After arrival at the laboratory, samples were placed in a -20 ${}^{\circ }$C freezer and analysed for glucocorticoid metabolites within 2 weeks. Faecal extracts were analysed using an enzyme immunoassay for 11β-hydroxyetiocholanolone, a group-specific assay for the measurement of cortisol metabolites with a 3α,11β-dihydroxy structure. The assay has previously been validated for monitoring glucocorticoid output in numerous species of primates (e.g. Heistermann et al., 2006), including Verreaux's sifakas (Fichtel et al., 2007). The assay was carried out as described in detail by Heistermann et al. (2004). Prior to assaying, all samples were diluted 1:400 in assay buffer (0.04 M PBS, pH 7.2), and duplicate 50 µL aliquots were taken to assay. Sensitivity of the assay at 90 % binding was 0.6 pg. Intra- and inter-assay coefficients of variation of high- and low-value quality controls were 6.5 % (high, n=16) and 8.4 % (low, n=16) and 7.1 % (high, n=6) and 12.5 % (low, n=6) respectively. All hormone concentrations are expressed as mass hormone per faecal mass (i.e. wet weight).

### 2.5 Data analyses

Differences in temperature between sites were estimated by fitting three linear mixed models (LMMs; Baayen, 2008) with either minimum, mean, or maximum temperature as response, site as fixed factor, and iButton ID as random factor. The influence of the distance to the edge border on temperature was estimated with three LMMs with either minimum, mean, or maximum temperature as response, distance of the iButton location to the edge border as fixed factor, and iButton ID as random factor. The models were fitted in R (R Core Team, 2015) using the lm function of the “lme4” R package (Bates et al., 2015).

Differences in time (rates per hour) spent travelling, feeding, resting, and interacting socially as well as time spent feeding on leaves, fruits, flowers, buds, and seeds between individuals occurring at the edge or interior forest were compared with a Mann–Whitney U test using Past3, as these data were not normally distributed.

ArcView (version 3.3) was used to calculate the home ranges of the study groups based on minimum convex polygons (MCPs). Adaptive kernel analyses were also used to calculate home ranges at 95 % of raster resolution and at 50 % for the core areas, using an automatically selected LCSV smoothing parameter (Burgman and Fox, 2003).

To test whether fGCM levels were influenced by study site (i.e. forest interior vs. edge habitat), we used a linear mixed model (Baayen, 2008) into which we included median fGCM levels as response, site as a fixed effect, and sex as control factor because some females were gestating. The model was fitted in R (R Core Team, 2015) using the lm function of the lme4 R package (version 3.3.2; Bates et al., 2015).

Groups in both habitats (edge: $N_{\mathrm{groups}}=3$; forest interior: $N_{\mathrm{groups}}=9$) are part of a long-term project in which group compositions including births have been assessed during regular census visits (Kappeler and Fichtel, 2012). Originally there were three groups of sifakas at the forest edge, but one group dissolved in 2011. To estimate differences in birth rates, we assessed whether each female (edge: $N_{\mathrm{females}}=4$; forest interior: $N_{\mathrm{females}}=12$) of each group gave birth (yes or no) between 2009 and 2013. We fitted a generalized linear mixed model (GLMM; Zuur et al., 2009) using the lme4 package (Bates et al., 2015) by including a binomial response term indicating whether or not a female gave birth in a given year and site as fixed factors as well as mother ID and year as random factors.

In general, after fitting each model, we checked the assumptions regarding the normality of distributions and homoscedasticity and compared the full model to a null model, including the control factor, using maximum likelihood ratio tests.

### 2.6 Ethical statement

This study was approved and authorized by the CAFF/CORE of the Direction des Eaux et Forêts Madagascar and the CNFEREF Morondava, Madagascar. Verreaux's sifakas (*Propithecus verreauxi*) are currently classified as critically endangered (IUCN, 2020).

## 3 Results

### 3.1 Sifaka densities

The study area at the edge comprised 31 ha and was inhabited by two groups consisting of six Verreaux's sifakas, whereas the study area in the forest interior comprised 70 ha and housed nine groups consisting of 44 individuals, indicating a lower population density in the edge habitat.

### 3.2 Ambient temperatures

Daily minimum, mean, or maximum temperatures did not differ between study sites (Fig. 2a–c; Table 2a–c). Daily mean temperatures decreased with incremental distance from the border of the edge towards the interior of the forest (Fig. 3, Table 2e). However, daily minimum and maximum temperatures did not change over distance (Table 2d, f).

**Table 2:** Parameter estimates from the linear mixed model (LMM) fitting differences in minimum, mean, and maximum temperature between study sites (a–c) and as a function of the distance to the edge border (d–f) with corresponding full–null model comparisons.

Response variable	Fixed factor	Estimate	SE	p value
(a) Minimum temperature	Intercept	14.26	0.16	<0.001
	Site	-0.22	0.22	0.337
Full–null model comparison: $\chi^{2}=1.05$, df=1, p=0.305				
(b) Mean temperature	Intercept	22.18	0.18	<0.001
	Site	-0.5	0.26	0.071
Full–null model comparison: $\chi^{2}=3.72$, df=1,p=0.054				
(c) Maximum temperature	Intercept	33.44	0.54	<0.001
	Site	-1.28	0.76	0.108
Full–null model comparison: $\chi^{2}=2.95$, df=1,p=0.086				
(d) Minimum temperature	Intercept	14.67	0.23	<0.001
	Distance	-0.002	0.001	0.066
Full–null model comparison: $\chi^{2}=4.45$, df=1,p=0.035				
(e) Mean temperature	Intercept	23.1	0.22	<0.001
	Distance	-0.004	0.001	0.001
Full–null model comparison: $\chi^{2}=13.932$, df=1,p<0.001				
(f) Maximum temperature	Intercept	34.89	1.32	<0.001
	Distance	-0.01	0.01	0.227
Full–null model comparison: $\chi^{2}=1.94$, df=1,p=0.164				

### 3.3 Home ranges

**Figure 1:**
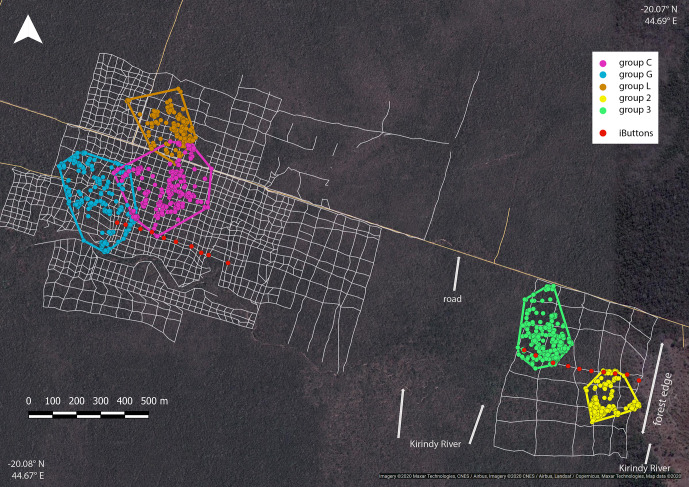
Satellite image of the study area depicting the home ranges of the study groups in the interior (groups C, G and L) and at the edge (groups 2 and 3) with MCPs (minimum convex polygons), the grid system of small foot trails (grey lines), and iButton locations (red dots). Imagery © 2020 Maxar Technologies, CNES/Airbus, Imagery © 2020/CNES/Airbus, Landsat/Copernicus, Maxar Technologies, Map data © 2020.

Home ranges varied in size between groups occurring at the edge and forest interior (Fig. 1; Table 3). Based on kernel analyses with 95 % and 50 % of raster resolution, groups at the edge had smaller home ranges (K95 %: 2.9, 3.1 ha; K50 %: 0.3, 0.1 ha) than groups in the forest interior (K95 %: 9.0, 9.8, 3.6 ha; K50 %: 02.9, 3.1 ha). Based on MCPs, only two groups in the interior had larger home ranges (9.4, 8.2, 4.7 ha) than the two groups at the edge (2.8 and 4.7 ha), and one group in each habitat type had about the same home range size.

**Figure 2:**
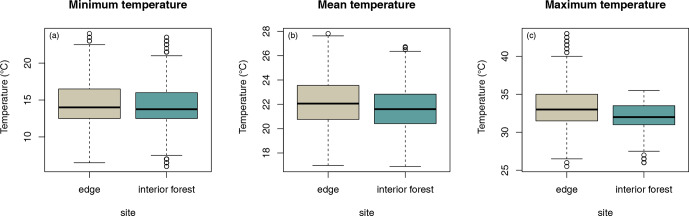
Differences in daily **(a)** minimum, **(b)** mean, and **(c)** maximum temperatures between sites.

**Figure 3:**
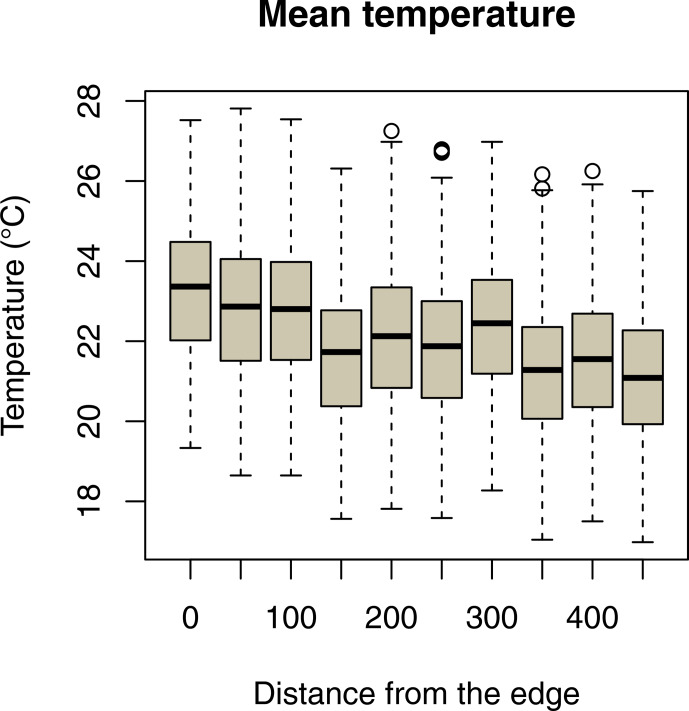
Decrease in daily mean temperatures as a function of the distance of iButton locations from the border of the forest edge.

**Table 3:** Results of the home range estimates of each group based on different methods.

Site	Group	Group	GPS	MCPs	K 95 %	Core area,	Intergroup
		size	points	(ha)	(ha)	K 50 % (ha)	encounters
Edge	2	2	144	2.8	2.9	0.3	8
	3	6	287	4.7	3.1	0.1	1
Interior	C	4	216	9.4	9.0	2.0	4
	G	4	216	8.2	9.8	1.6	0
	L	3 (4)	152	4.7	3.6	0.4	1

MCPs denotes minimum convex polygons, and K denotes kernel analysis.

### 3.4 Activity budgets

**Figure 4:**
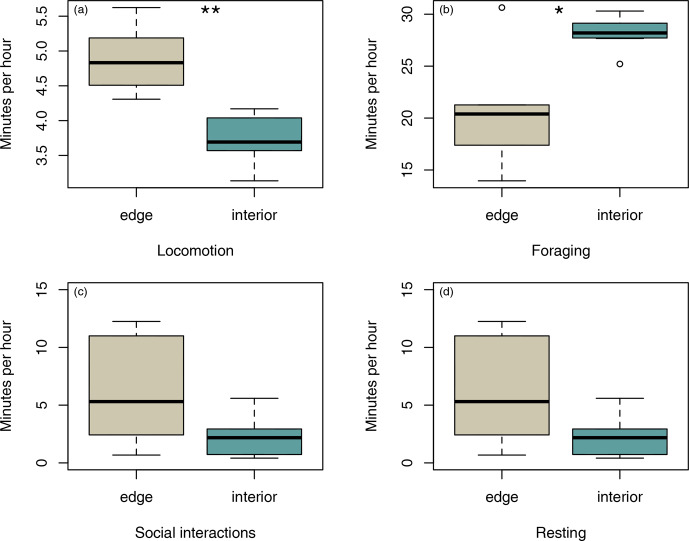
Differences in average duration of **(a)** locomotion, **(b)** foraging, **(c)** social interactions, and **(d)** resting between groups at the two study sites. Depicted are medians (black horizontal lines), interquartile ranges (boxes), and outliers (circles).

Sifakas near the forest edge spent less time feeding (Fig. 4a; Mann–Whitney U test, U=8, p=0.045) but more time travelling than sifakas inhabiting the forest interior (Fig. 4b; Mann–Whitney U test, U=0, p=0.002). However, the two populations did not differ with respect to average time spent socializing or resting (Fig. 4c, d; Mann–Whitney U test: socializing: U=11, p=0.107; resting: U=14, p=0.22).

### 3.5 Consumed feeding plants

**Figure 5:**
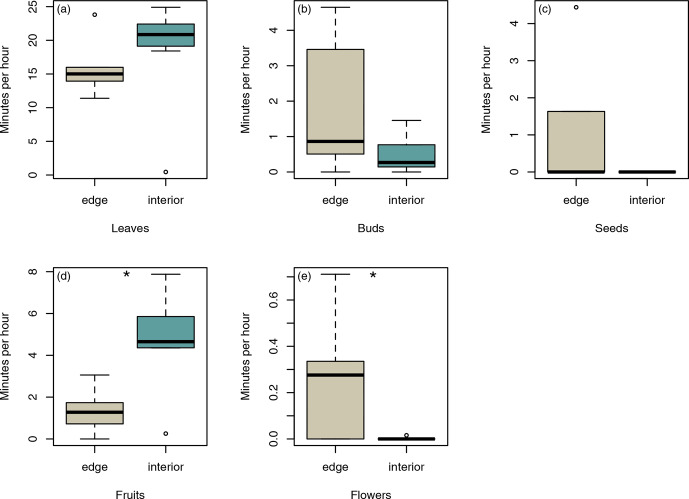
Differences in average time spent feeding on **(a)** leaves, **(b)** buds, **(c)** seeds, **(d)** fruits, and **(e)** flowers. Depicted are medians (black horizontal lines), interquartile ranges (boxes), and outliers (circles).

In total, sifakas consumed leaves, fruits, flowers, buds, and seeds of 45 different plant species (edge: 22 species; interior: 32 species). Sifakas at the forest edge fed on all items, whereas sifakas in the forest interior fed mainly on leaves, fruits, and flowers; however, the average feeding times on these food items did not differ significantly between populations (Fig. 5a, b, c; Mann–Whitney U test: leaves: U=12, p=0.138; buds: U=14.5, p=0.244; seeds: U=16, p=0.112). However, sifakas in the interior spent more time feeding on fruits, whereas sifakas at the edge spent more time feeding on flowers (Fig. 5c, d; Mann–Whitney U test: fruits: U=5, p=0.017; flowers: U=9, p=0.029).

The five most often consumed species varied between the two populations, with sifakas at the edge consuming primarily Namologna (*Foetidia retusa*) and Alimboro (*Albizia bernieri*), whereas sifakas in the forest interior consumed primarily Manjakabenitany (*Baudouinia fluggeiformis*) and Anakaraky (*Dupuya madagascariensis*). Feeding tree characteristics, such as dbh, tree height, and crown diameter, did not differ between study sites (Mann–Whitney U test: dbh: p=0.21; height: p=0.08; crown diameter: p=0.13).

### 3.6 Variation in fGCM concentrations

**Figure 6:**
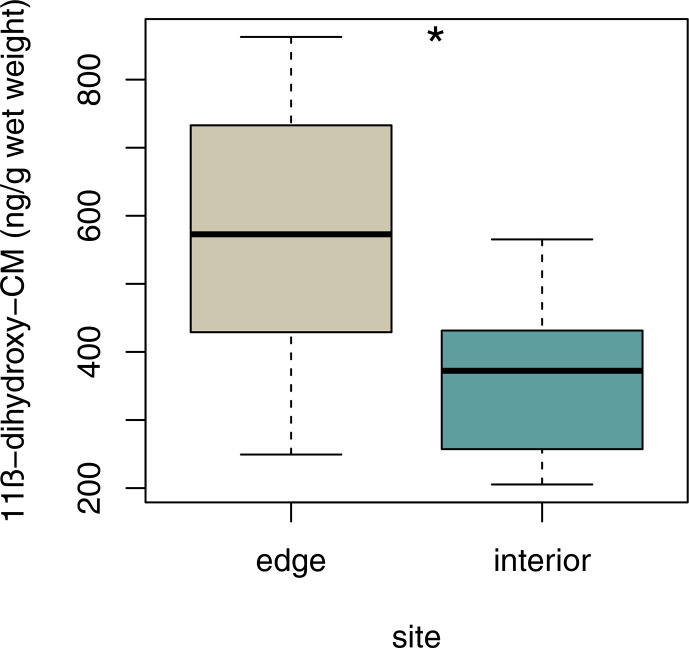
Median faecal glucocorticoid metabolite concentrations for each study site. Depicted are medians (black horizontal lines) and interquartile ranges (boxes).

Sifakas at the edge had higher average fGCM concentrations than those inhabiting the forest interior (Fig. 6; LMM, full–null model comparison: $\chi^{2}=5.59$, df=1, p=0.038; site: estimate ± SE: -212.43±89.83, p=0.038; sex: estimate ± SE: -97.58±92.78, p=0.316).

### 3.7 Reproductive rates

**Figure 7:**
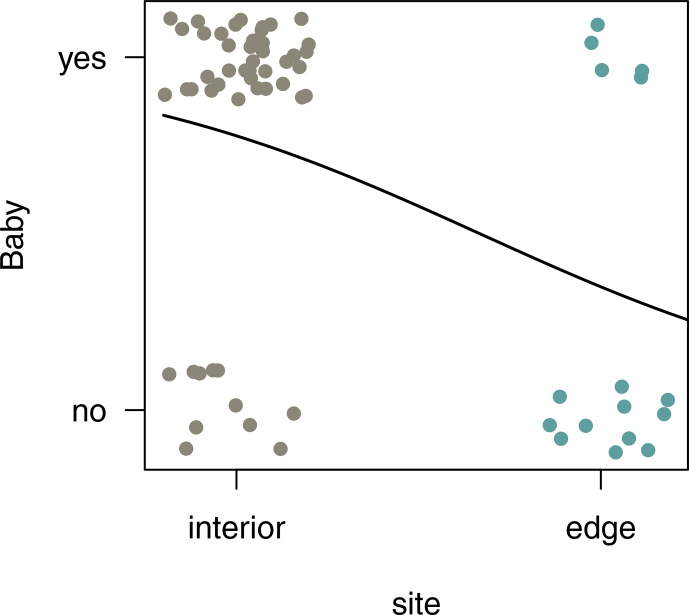
Probability (yes or no) of giving birth for females living in the edge or forest interior habitat.

In both habitats, each group included only one adult female. Of the two females in the edge population, only one gave birth, whereas in the three groups living in the forest interior, all females gave birth a month after the completion of this study. To compare reproductive rates, we analysed additional demographic data from 4 females from three different groups at the edge and 12 females from nine different groups in the interior. Sifakas near the edge had significantly lower birth rates compared with those living in the forest interior. Based on 45 infants born at both sites between 2009 and 2013, the average birth rate per female was 0.31 infants per year at the edge and 0.78 infants per year in the interior. The probability that a female gave birth was influenced by site but not by year (Fig. 7; GLMM, full–null model comparison: $\chi^{2}=11.77$, df=1, p<0.001; site: estimate ± SE: -2.08±0.643, p=0.001).

## 4 Discussion

The results of this study indicate that Verreaux's sifakas may respond behaviourally and physiologically to habitat variation related to the forest edge, which differs with respect to forest structure (Rakotoniaina et al., 2016) and average temperatures close to the border of the edge but not with respect to crude measures of feeding tree characteristics. Sifakas living in the edge habitat appeared to have lower population densities and smaller home ranges, but they spent more time travelling. In addition, they spent less time feeding but did not differ from the interior groups regarding time spent socializing and resting. Both populations had broadly similar diets, but only sifakas in the edge habitats consumed seeds and fed less often on fruits but more often on flowers. These behavioural differences appear to be biologically meaningful because sifakas living near the forest edge had elevated fGCM concentrations and lower birth rates. Altogether, these preliminary results based on a small sample size suggest that Verreaux's sifakas in Kirindy Forest may exhibit a negative edge response.

The microclimate in the edge habitat in Kirindy Forest did not differ with respect to daily temperatures from the forest interior, but it exhibited higher daily mean temperatures directly at the border of the habitat. The edge habitat was, similarly to other edge habitats in Madagascar, characterized by a lower tree density (Lehtinen et al., 2003; Lehman et al., 2006a–c; Rakotoniaina et al., 2016). Despite the lower tree density, both habitats exhibit broadly similar additional characteristics, including traversal by a river and a dirt road. However, sifakas at the edge inhabited substantially smaller home ranges than groups in the interior; in fact, they represent the smallest known home ranges of Verreaux's sifakas (Carrai and Lunardini, 1996; Richard, 1978; Benadi et al., 2008; Koch et al., 2016). Moreover, home ranges of groups in the interior forest overlapped among groups, whereas in the edge habitat, the two groups were separated by an area that was not occupied by sifakas. Whereas two groups of sifakas in the forest interior regularly crossed the dirt road, the home ranges of the groups living at the forest edge did not include the dirt road, and the home range of one group at either site bordered on the riverbed (Fig. 1). Therefore, we believe that potential effects derived from the dirt road and/or the river are minor at best with respect to causing the inter-site behavioural and physiological differences seen between the populations. Further, without data on habitat quality within home ranges, it is difficult to assess potential difference between the populations, but the higher travelling activity and fGCM levels indicate that the edge habitat might have been of lesser quality, despite the potential benefit of additional light and plant productivity (Ganzhorn, 1992).

Interestingly, the closely related Coquerel's sifakas in dry deciduous forests in Ankarafantsika National Park exhibited the opposite pattern, with edge groups inhabiting larger home ranges than groups in the forest interior (McGoogan et al., 2009). Activity budgets and food quality did not differ between those habitats, but larger home ranges of edge groups might have been due to the fact that these Coquerel's sifakas did not use the immediate forest edge and instead roamed about 400 m away from it. Similar, Diademed sifakas in fragmented habitats avoided the edges, whereas those in continuous forests did not avoid the edge (Irwin, 2008). In our study, where the edge presents a naturally derived edge, one group of Verreaux's sifakas inhabited a home range that encompassed the border of the edge and, hence, did not appear to avoid the edge.

The five most commonly consumed feeding trees of sifakas did not differ in size between sites, and both populations had broadly similar diets. Groups in the forest interior, however, spent more time feeding on fruits, whereas edge groups spent more time feeding on seeds and flowers, which are more quickly ingested than fruits and may require higher rates of switches between feeding sites (Norscia et al., 2006; Irwin, 2008; Gabriel, 2013). This fact may explain why edge-living sifakas spent more time travelling than individuals in the forest interior. As we only have phenology data for the interior forest, we cannot evaluate whether there were generally fewer fruiting trees present in the edge habitat. The presence of the Kirindy River, which runs through both habitats, however, should have a similar impact on tree phenology at both sites.

Nevertheless, the higher fruit feeding rates of the interior groups may suggest that they may have enjoyed a higher energy intake. Reduced energy intake via reduced fruit consumption is often associated with an increase in glucocorticoid output, indicating nutritional stress (Creel et al., 2002; Homan et al., 2003; Pride, 2005; Dunn et al., 2013). In chimpanzees (*Pan troglodytes*), for example, urinary cortisol levels have been found to increase when they feed less on fruits (Muller and Wrangham, 2004). Similarly, fGCM levels have been reported to be negatively correlated with habitat quality in Diademed sifakas (*P. diadema*) as well as in both Diademed and Verreaux's sifakas during periods of fruit scarcity (Tecot et al., 2019; Rudolph et al., 2020). Moreover, energetically costly travelling rates are associated with an increase in fGCM levels in mantled howler monkeys (*Alouatta palliata*; Gómez-Espinosa et al., 2014). However, daily travel distances and variation in home range size did not affect fGCM levels in Verreaux's sifakas living in the forest interior (Rudolph et al., 2019). Hence, increased fGCM levels found in the present study might be due to the lower energy intake, i.e. the lower rate of fruit consumption, by sifakas inhabiting the edge habitat.

In contrast to faecal glucocorticoid measurements, hair cortisol measurements integrate changes in glucocorticoid output over periods of weeks and months and may provide information on chronically increased stress hormone levels. Squirrel gliders (*Petaurus norfolcensis*) have been found to exhibit higher hair cortisol levels in urbanized edges than in intact habitats (Brearley et al., 2012). However, edge-positive grey mouse lemurs (*Microcebus murinus*) and edge-negative fat-tailed dwarf lemurs (*Cheirogaleus medius*), which were studied in the same edge and interior forest habitat as in our study, did not differ in hair cortisol levels and parasite burden (Rakotoniaina et al., 2016). As glucocorticoid concentrations in sifakas in our study were measured in faecal samples reflecting stress hormone output over a shorter timescale (Fichtel et al., 2007), our fGCM measure might more likely reflect a response to short-term variation in diet rather than a general adaptation to living in the edge habitat. Hence, measurements of sifaka hair cortisol are required to confirm this assumption. In this respect, in eastern chipmunks (*Tamias striatus*) individuals from logged habitats have been found to have higher faecal glucocorticoid metabolite levels than individuals from intact habitats, but hair cortisol levels did not differ between these groups (Mastromonaco et al., 2014). As chipmunks could easily move between logged and undisturbed areas and faecal samples were collected from the periphery of the logged area, the increase in faecal glucocorticoid metabolite levels might rather reflect short-term increases in cortisol, whereas the similar hair cortisol levels of individuals in both areas may indicate that habitat disturbance may not have affected long-term stress hormone output. Hence, depending on the glucocorticoid measure taken, i.e. from faeces or hair, the temporal scale of the sample collection should be taken into account (Mastromonaco et al., 2014).

At the ultimate level, elevated glucocorticoid concentrations are often associated with impairment of gonadal function and reproductive behaviour in female and male vertebrates (Cameron, 1997; Hardy et al., 2005) and are linked to reduced reproductive output in a variety of species (e.g. Ellenberg et al., 2007; Buck et al., 2007; Dantzer et al., 2014; Vitousek et al., 2018). For example, in northern saw-whet owl (*Aegolius acadicus*) habitat loss and fragmentation increased physiological stress levels in males and resulted in lower reproductive success (Hinam and St. Clair, 2008). Here, our data suggest that such a link between elevated glucocorticoid levels and decreased reproductive success may also exist for edge-living Verreaux's sifakas, which, according to our long-term records, show significantly reduced birth rates when compared with sifakas living in the forest interior. However, the small number of animals studied for stress hormone output and the fact that our faecal measure of glucocorticoid output may not fully reflect the effect of long-term stress precludes us from drawing firm conclusions. Future studies including more animals possibly in other edge-habitat locations and GCM analyses from faeces over a more extended period or even cortisol analyses from hair should be carried out to substantiate our preliminary reasoning. These future studies may also try to collect comparative data on female ovarian activity for assessing ovulation and conception rates, reproductive behaviour, and possibly energetic condition to help uncover the possible mechanisms underlying a potential link between stress hormone output and reproductive function and success in Verreaux's sifaka populations living in different habitat types. This, in turn, may help improve the conservation efforts for this highly endangered primate species.

## 5 Conclusions

Although Verreaux's sifakas in Kirindy survive and reproduce in the edge habitat, they appear to be sensitive to variation in microhabitat conditions that may be linked to the forest edge. However, more information on habitat characteristics in the edge and interior forest is required to test this notion. As the forest cover in the Kirindy Forest region continues to decline (Zinner et al., 2014), and habitat fragmentation into smaller forest patches leads to a decrease in the interior-to-edge ratio, the results of this study – indicating that Verreaux's sifakas might show a negative edge response – offer relevant information for future assessments of the conservation status of this species.

## Supplement

10.5194/pb-8-1-2021-supplement The supplement related to this article is available online at: https://doi.org/10.5194/pb-8-1-2021-supplement.

## Data Availability

Data are available in the Supplement.
