# Supplementary material for: Life on the edge: behavioural and physiological responses of Verreaux's sifakas (Propithecus verreauxi) to forest edges
Source: Primate Biol. 2021 Feb 9;8(1):1–13. doi: 10.5194/pb-8-1-2021 (PMC8129909; doi:10.5194/pb-8-1-2021)
Supplement: The supplement related to this article is available online at: https://doi.org/10.5194/pb-8-1-2021-supplement. [file pb-8-1-supplement.pdf]

Supplement of Primate Biol., 8, 1–13, 2021
https://doi.org/10.5194/pb-8-1-2021-supplement
© Author(s) 2021. This work is distributed under
the Creative Commons Attribution 4.0 License.

Primate Biology
Open Access

Supplement of

Life on the edge: behavioural and physiological responses of Verreaux's sifakas (*Propithecus verreauxi*) to forest edges

Klara Dinter et al.

Correspondence to: Claudia Fichtel (claudia.fichtel@gwdg.de)

The copyright of individual parts of the supplement might differ from the CC BY 4.0 License.

Supplementary material:

Table 1: Min and max temperatures in celsius

min edge	max edge	min interior	max interior
20,5	38,5	20	35
21	38,5	21	34
20	41,5	20	35,5
19	39,5	18,5	35
15,5	40	15	34,5
14,5	42	14,5	34,5
14,5	41,5	14	34
12,5	41,5	12	34,5
13	41,5	13	34,5
13	43	12,5	35
13	42,5	13	35
16,5	42	16,5	35
17,5	43	17,5	35,5
17	39,5	17	35
16	42	16	35
13,5	41	13,5	34,5
12	42,5	12	35
16	42	16	35
18	42	18	35,5
17	42	17	35
18	41,5	17	35
15,5	40	15,5	34
15,5	39	15,5	33
12,5	37,5	12	31,5
11	36,5	10	30,5
13,5	38,5	12,5	31,5
11	37	10,5	31
13,5	34,5	13	31,5
13	37	13	33
16	35	15,5	31,5
13	34,5	12,5	30
16,5	37	16,5	32,5
14,5	35,5	13,5	31,5
13,5	38,5	13,5	34
14	39,5	13,5	35
18	41	17,5	35,5
19,5	40,5	19	35
15,5	39,5	15,5	34
13,5	40	13,5	34

15,5	39,5	15	33,5
13,5	39,5	13	32
13	37,5	12	31,5
12,5	36,5	11,5	30,5
9,5	36,5	9	31
9	38,5	9	33
7	38,5	6,5	32
7,5	38,5	7,5	32,5
10	39,5	10	33,5
13,5	38,5	13	33
15	35	14,5	32,5
12,5	34,5	12,5	31
13,5	39,5	13	34
16,5	36,5	16	32
16	37,5	16	33
13	39	13	33,5
12	39	12	33,5
12,5	39	12,5	34
12,5	40	12	34,5
10,5	40	11,5	34,5
13,5	39,5	14	34,5
10,5	38	11	33,5
12,5	40,5	12,5	34,5
12	38	11,5	33,5
14	38,5	13,5	32,5
15	38	15	32,5
16,5	37	16	33,5
14,5	37	14	33
14	37,5	14,5	31,5
13	36,5	12,5	31,5
13	37	12,5	32
10,5	38	10,5	32,5
10	38,5	9,5	34
10	37,5	9,5	34
13,5	39,5	13,5	34
10,5	38	10	33
7	38,5	6,5	34
8	37	7,5	34,5
7	38	6,5	33,5
6,5	38	6	34

Table 2: Behavioral activities (sec/hr)

site	social	locomotion	resting	feeding
edge	12,17	77,53	435,73	551,52
edge	94,6	81,15	528,55	370,58
edge	197,95	101,23	524,37	251,28
edge	96,45	93,38	526,6	363,57
edge	43,63	85,3	563,15	382,78
edge	220,45	88,65	456,43	312,97
interior	17,33	75,05	487,93	499,68
interior	7,42	64,97	472,2	535,42
interior	8,8	66,48	536,9	453,77
interior	36,3	71,97	459,63	506,78
interior	50,38	63,52	415,57	545,47
interior	42,23	56,43	473,05	508,28
interior	55,32	66,43	440,07	513,43
interior	100,65	73,43	405,38	497,95

Table 3: Time spent feeding on different food items (min/hr)

site	food_item	duration	food_item	duration	food_item	duration
edge	buds	0,00	flowers	0,00	fruits	0,00
edge	buds	0,51	flowers	0,34	fruits	0,72
edge	buds	0,82	flowers	0,25	fruits	1,09
edge	buds	0,90	flowers	0,30	fruits	1,47
edge	buds	3,46	flowers	0,71	fruits	1,74
edge	buds	4,65	flowers	0,00	fruits	3,06
interior	buds	0,00	flowers	0,00	fruits	0,26
interior	buds	0,14	flowers	0,00	fruits	4,36
interior	buds	0,14	flowers	0,00	fruits	4,36
interior	buds	0,15	flowers	0,00	fruits	4,63
interior	buds	0,38	flowers	0,00	fruits	4,68
interior	buds	0,52	flowers	0,02	fruits	5,38
interior	buds	1,02	flowers	0,00	fruits	6,33
interior	buds	1,46	flowers	0,00	fruits	7,88

site	food_item	duration	food_item	duration
edge	leaves	11,39	seeds	0,00
edge	leaves	13,94	seeds	0,00
edge	leaves	14,18	seeds	0,00
edge	leaves	15,84	seeds	0,00
edge	leaves	15,99	seeds	1,63
edge	leaves	23,81	seeds	4,44
interior	leaves	0,45	seeds	0,00
interior	leaves	18,42	seeds	0,00
interior	leaves	19,84	seeds	0,00
interior	leaves	20,11	seeds	0,00
interior	leaves	21,59	seeds	0,00
interior	leaves	22,12	seeds	0,00
interior	leaves	22,70	seeds	0,00
interior	leaves	24,90	seeds	0,00

Table 4: Tree characteristics

tree species (vernacular name)	dbh (cm)	height (m)	crown dia (m)	site
Tratramborondreo	16,71	16,5	10	edge
Mandakolahy	11,46	19	8	edge
Namologna	10,19	9	5,5	edge
Laro	7,64	7	4,5	edge
Namologna	7	8	5,5	edge
Alimboro	8,75	8,5	4	edge
Tratramborondreo	6,53	7	3	edge
Vahymainty	NA	10	NA	edge
Tsilaitsy	4,46	4,5	3	edge
Refeko	4,62	6,5	1	edge
Alimboro	9,87	8	4	edge
Karimbolavahy	NA	10	NA	edge
Selybe	7,96	4	3	edge
Namologna	10,5	14	5	edge
Namologna	15,6	17	7	edge
Kily	19,1	15	14	edge
Tratramborondreo	14,32	12	6	edge
Alimboro	12,1	6	8	edge
Alimboro	11,62	11,5	5	edge
Laro	8,59	7,5	4	edge
Mendoravy	6,68	8,5	2	edge
Mendoravy	20,05	18	9	edge
Kily	25,94	17	18	edge
Tahambyo	2,23	5	1	edge
Nato kironono (gr. f.)	7,32	8	7	edge
Manjakabenitany	8,28	7	4	edge
Alimboro	17,83	17	8	edge
Manjakabenitany	15,92	12	15	edge
Alimboro	18,46	18	9	edge
Alimboro	11,46	11,5	4,5	edge
Alimboro	8,75	11	4	edge
Namologna	8,28	7	4	edge
Sarongaza	24,19	19	7	edge
NA	9,87	17	5	edge
Alimboro	18,3	18	9	edge
Nato kironono (gr. f.)	7	9	4,5	edge

Alimboro	20,37	16	11	edge
Tratramborondreo	16,71	15	7	edge
Namologna	10,66	9,5	4,5	edge
Hazompasy	8,75	7,5	5	edge
Karimbolavahy	NA	10	NA	edge
Selivoloe	6,05	10	5	edge
Alimboro	10,19	12,5	5,5	edge
Namologna	8,28	8,5	3,5	edge
Vahypihindy	NA	5	NA	edge
Manjakabenitany	9,39	8	4,5	edge
Hazolandiala	7,8	11	2,5	edge
Karimbolavahy	NA	8	NA	edge
Alimboro	11,14	13	5	edge
Hazomena	6,21	7	2,5	edge
Namologna	10,5	12	4	edge
Laro	4,77	6	1	edge
Namologna	15,92	11,5	5,5	edge
Talafoty	5,73	9	3	edge
Namologna	12,25	11	4,5	edge
Tratramborondreo	7,16	10	4	edge
Manjakabenitany	3,02	5,5	1	edge
Valotsy	14,64	12	7	edge
Vahymainty	NA	10	NA	edge
Alimboro	9,87	11	4	edge
Namologna	20,69	14	5,5	edge
Alimboro	15,92	12	7	edge
Kily	14,48	7	7	edge
Namologna	8,75	10	3	edge
Alimboro	12,89	11	6	edge
Refeko	1,91	3,5	1	edge
Namologna	6,84	8,5	2,5	edge
Namologna	9,07	9	4,5	edge
Manjakabenitany	10,19	10	5,5	edge
Alimboro	5,57	6,5	2,5	edge
Namologna	8,28	7	3	edge
Vahymainty	NA	7	NA	edge
Kitakitakala	6,05	6	6	interior
Sarisakoambanditry	4,93	6	2	interior

Hazomby	4,14	6	3,5	interior
Nato	11,14	10	4	interior
Kitakitakala	4,77	4,5	3	interior
Sarisakoambanditry	7,96	7,5	3,5	interior
Sarongaza	23,87	18	12	interior
Anakaraky	26,72	12	10	interior
Sarongaza	25,86	17,5	10	interior
Mangaryfotry	10,03	15	6	interior
Anakaraky	25,62	14	10	interior
Tsylavonrya	5,57	4	2,5	interior
Selyvolohe	3,34	5	1,5	interior
Maintifototry	9,71	6	3,5	interior
Sarisakoambanditry	5,25	6	2,5	interior
Manjakabenitany	15,92	13,5	10	interior
Hazompasy	12,1	15	5	interior
Tratramborondreo	11,3	14	6	interior
Hazomby	4,46	4,5	4	interior
Maintifototry	4,77	6,5	2,5	interior
Maintifototry	4,3	4,5	2	interior
Tratramborondreo	3,02	4,5	2,5	interior
Manjakabenitany	14,01	8	2,5	interior
Maintifototry	5,25	7	3	interior
Manjakabenitany	8,28	7	3	interior
Namologna	12,65	9,5	5	interior
Sahandraha	10,5	18	4	interior
Maintifototry	6,53	9	2	interior
Maintifototry	6,37	7	4	interior
Manjakabenitany	13,37	12,5	5	interior
Mendoravy	15,52	12,5	5	interior
Sarongaza	22,76	22	10	interior
Manjavasarotsy	3,66	5	2,5	interior
Kitakitakala	6,37	4	3	interior
Manjakabenitany	10,5	9	5	interior
Anakaraky	29,2	15	15	interior
Menavahatsy	6,05	7	3	interior
Manjavasarotsy	6,37	4	2	interior
Manjakabenitany	10,98	7	4	interior
Ampoly	3,18	4	2	interior

Fony	31,83	18	6	interior
Alimboro	10,35	9	9	interior
Tratramborondreo	10,98	13	5	interior
Menavahatsy	4,3	7	4,5	interior
Tanjaky	4,62	7,5	7	interior
Maintifototry	7,64	5	2	interior
Tratramborondreo	4,14	7	3	interior
Menavahatsy	3,5	7	3	interior
Maintifototry	4,77	7	1,5	interior
Manjavasarotsy	3,02	5	2,5	interior
Sarongaza	16,87	20	9	interior
Mandakolahy	9,71	19	6,5	interior
Manjakabenitany	10,82	8	4	interior
Tratramborondreo	7,48	5	2	interior
Ampoly	5,57	4,5	3	interior
Menambaho	12,1	16	5,5	interior
Menambaho	11,3	18	6	interior
Tsiandala	10,03	9	3	interior
Menavahatsy	3,34	5	1,5	interior
Manjakabenitany	14,32	7,5	3	interior
Maintifototry	3,66	5,5	1,5	interior
Tratramborondreo	5,25	9	2	interior
Mansavasarotsy	3,98	5	2	interior
Tsiandala	9,87	12	4,5	interior
Sarihas	NA	2,5	NA	interior
Mansavasarotsy	4,14	5,5	2	interior
Manjakabenitany	15,12	9,5	4	interior
Maintifototry	3,66	5	2	interior
Alimboro	5,41	7	3,5	interior
Hazompasy	5,57	4	2,5	interior
Menambaho	16,71	17	6	interior
Fony	49,82	15	8,5	interior
Mansavasarotsy	4,77	7	2,5	interior
Teloravy	NA	5	NA	interior
Ampoly	3,5	6	1,5	interior
Manjakabenitany	18,62	10	8	interior
Manjakabenitany	17,19	8,5	5	interior
Anakaraky	35,33	16,5	13	interior

Anakaraky	20,45	17	8	interior
Fony	46,79	15,5	8,5	interior
Menambaho	6,84	13	3	interior
Manjakabenitany	13,37	7	3	interior
Tratramborondreo	7,8	10	5	interior
Fony	34,54	18	6	interior
Mansavasarotsy	3,34	5	1,5	interior
Menambaho	16,71	16,5	6	interior
Mandakolahy	9,23	14	5	interior
Menavahatsy	4,46	8	2,5	interior
Fony	54,91	17	9	interior
Fony	56,5	17	10	interior
Menavahatsy	3,34	6	1,5	interior
Manjakabenitany	17,35	10	7	interior
Anakaraky	23,71	13	12	interior
Maintifototry	13,21	12	9	interior
Menambaho	14,01	11	8,5	interior
Manjakabenitany	8,28	9	4,5	interior
Tsiandala	10,35	9	5,5	interior
Maintifototry	6,05	5,5	4	interior
Menambaho	12,1	14	7	interior
Manjakabenitany	17,51	10	5,5	interior
Tratramborondreo	9,55	12	5	interior
Namologna	7,96	8,5	3	interior
Mandravasotra	4,62	5,5	2	interior
Karimbolavahy	NA	2,5	NA	interior
Menavahatsy	3,66	7	3	interior
Tanjaky	4,3	7	3,5	interior
Tsiandala	13,05	8	4	interior
Menavahatsy	1,59	2,5	1	interior
Menambaho	12,41	18	5,5	interior
Menambaho	14,01	18	6	interior
Mansavasarotsy	2,55	4	1,5	interior
Mansavasarotsy	7	6,5	2,5	interior
Tsiandala	13,21	8	5	interior
Manjakabenitany	9,71	9	4,5	interior
Maintifototry	10,35	11	5,5	interior
Vahypinty	NA	7	NA	interior

Mansavasarotsy	7,16	8	4	interior
Sarihompy	8,59	7,5	3,5	interior
Relambo (pt.fl.)	3,98	7	3	interior
Nato	10,35	11	3,5	interior
Mansavasarotsy	3,18	7,5	1	interior
Fony	65,25	18	12	interior
Mansavasarotsy	6,53	7	2,5	interior
Mandravasotra	6,68	6,5	3,5	interior
Manjakabenitany	12,73	8	6,5	interior
Menambaho	15,76	18	8,5	interior
Manjakabenitany	12,41	11	5,5	interior
Tsilaiby	14,32	12	12	interior

Table 5: median FGC metabolite concentrations

site	sex	median fGC
edge	f	732,89
edge	m	249,26
edge	f	578,84
edge	m	566,83
edge	m	862,7
edge	m	428,99
interior	f	405,25
interior	m	259,11
interior	m	205,31
interior	f	255,28
interior	m	382,4
interior	m	362,17
interior	f	565,32
interior	m	457,6

Table 6: Birth rates from 2009-2013

site	gave birth (0=no, 1=yes)	year	mother_ID
edge	0	2009	1
edge	0	2009	2
edge	0	2009	3
edge	1	2009	4
edge	0	2010	1
edge	0	2010	2
edge	0	2010	3
edge	0	2010	4
edge	0	2011	1
edge	0	2011	2
edge	0	2011	3
edge	1	2011	4
edge	1	2012	3
edge	1	2012	4
edge	1	2013	3
edge	0	2013	4
interior	1	2009	5
interior	0	2009	6
interior	1	2009	7
interior	0	2009	8
interior	1	2009	9
interior	1	2009	10
interior	1	2009	11
interior	1	2009	12
interior	1	2009	13
interior	1	2009	14
interior	1	2009	15
interior	1	2009	16
interior	1	2010	5
interior	1	2010	6
interior	1	2010	7
interior	1	2010	8
interior	0	2010	9
interior	1	2010	10
interior	0	2010	11
interior	1	2010	13
interior	0	2010	14
interior	0	2010	15

interior	1	2010	16
interior	0	2011	6
interior	1	2011	7
interior	0	2011	8
interior	1	2011	9
interior	1	2011	10
interior	1	2011	11
interior	0	2011	13
interior	1	2011	14
interior	1	2011	15
interior	1	2011	16
interior	1	2012	6
interior	1	2012	8
interior	1	2012	9
interior	1	2012	10
interior	1	2012	11
interior	1	2012	13
interior	1	2012	14
interior	1	2012	15
interior	1	2012	16
interior	1	2013	6
interior	0	2013	8
interior	1	2013	9
interior	1	2013	10
interior	1	2013	11
interior	1	2013	13
interior	0	2013	14
interior	1	2013	15
interior	1	2013	16
